# Deserts, Swamps and Food Oases: Mapping around the Schools in Bahia, Brazil and Implications for Ensuring Food and Nutritional Security

**DOI:** 10.3390/nu16010156

**Published:** 2024-01-03

**Authors:** Fabiana Chagas Oliveira de França, Renata Puppin Zandonadi, Iana Mendes de Almeida Moreira, Izabel Cristina Rodrigues da Silva, Rita de Cassia Coelho de Almeida Akutsu

**Affiliations:** 1 Nutrition School, Federal University of Bahia-Augusto Viana, s/n-Palácio da Reitoria, Canela, Salvador 40110-907, Brazil; fabianacofranca@hotmail.com; 2 Department of Nutrition, University of Brasília, Brasília 70910-900, Brazil; renatapz@unb.br; 3 Faculdades AGES de Jacobina, Avenida Universitária, 701-Bairro Pedra Branca, Jacobina 44700-000, Brazil; ianamendesmoreir@gmail.com; 4 Faculty of Ceilândia, University of Brasília, Brasília 72220-275, Brazil; belbiomedica@gmai.com

**Keywords:** food deserts, food swamps, food oases, schools, adolescents

## Abstract

Deserts, swamps and food oases terms have been used to characterize the food environment according to the identified food availability. Food swamps are defined as neighborhoods with a predominance of food establishments selling ultra-processed foods compared to establishments selling healthy options. In contrast, food oases are areas with easy access to healthy and nutritious foods, such as fruits, vegetables, and other fresh foods. Food deserts describe densely populated urban areas where residents face difficulty obtaining healthy food. In this context, this work aimed to map deserts, swamps, and food oases around federal schools in Bahia, Brazil, emphasizing the importance of implementing the Brazilian National School Feeding Program (PNAE) in these schools, to guarantee food security and nutrition. An ecological study was carried out in all 35 federal schools in Bahia, Brazil, using an 800 m buffer analysis, with the school as the centroid. The geographic coordinates of schools and food establishments were initially obtained using Google Maps and later confirmed onsite. To evaluate food deserts and swamps, the methodology proposed by the CDC was used and the Modified Retail Food Environment Index (mRFEI) was calculated; when the result was equal to zero, the surrounding area was considered a food desert and values between 0.01 and 20 determined food swamps; for values above 20, the neighborhood was classified as mixed. Food oases were considered regions containing at least one supermarket within the analyzed buffer. Descriptive analyses were carried out with frequency measurements, measures of central tendency (mean and median) and dispersion (standard deviation). The food environment of schools was compared considering the number of students impacted, the area where the school was located (urban or rural) and the size of the municipalities. The average number of food establishments found was 22.39 (±13.03), with the highest averages for snack bars (7.33 ± 4.43), grocery stores (5.83 ± 4.09) and restaurants (2.94 ± 2.19). Food deserts and mixed environments were identified in 40% of the sample, while swamps represented 20% and oases 65%. An association was observed between food deserts and social vulnerability, making it necessary to emphasize the importance of adequate implementation of the PNAE in these schools to reduce food and nutritional insecurity, guaranteeing the human right to adequate and healthy food and providing better nutrition and health perspectives within the school environment and impact on students’ lives through food and nutrition education actions, which are also part of the context of PNAE activities.

## 1. Introduction

The contemporary model that adopts hegemonic food systems, focusing on quantity over food quality, has been the subject of essential discussions about the food environment and the negative impacts on students’ health, especially concerning poor nutrition, obesity, and health harms it can cause [1,2,3,4]. As a challenge, it is necessary to consider the dimensions related to inequality in physical access and distribution of food, losses, and waste, unsustainable production and consumption, which generally have a greater impact on vulnerable populations, such as children and adolescents [5,6,7].

In recent years, research on food environments has increased. The studies started mapping the availability of food in neighborhoods, communities, and municipalities and evolved to the analysis of school neighborhoods, seeking to understand to what extent the characterization of these environments, considering the type of food available, plays a determining role in food choices and the nutritional profile of the adolescents [1,8,9,10,11].

In food environment studies, several terms have been used to define spaces based on the number of establishments and the type of food available in these environments, with food deserts being the most discussed, followed by swamps and food oases, among others less explored [12,13].

The term food desert has been highlighted recently. It describes densely populated urban areas where residents face difficulties obtaining healthy food. In Brazil, the *Câmara Interministerial de Segurança Alimentar e Nutricional* (CAISAN) conducted the “Technical Study: Mapping of Food Deserts in Brazil” to map and analyze in detail the food retail trade in the country. In this study, food deserts were defined according to the physical dimension of access, related to the proximity between homes and establishments where it is possible to purchase healthy food [14].

Food swamps are defined as neighborhoods with a predominance of food establishments selling ultra-processed foods compared to those selling healthy options [13,15,16,17]. In contrast, food oases are areas with easy access to healthy and nutritious foods, such as fruits, vegetables, and other fresh foods. These areas often have various healthy food options in supermarkets, grocery stores, and other retail establishments [12]. These concepts aim to offer an analysis of the environments, connecting them to the determining variables of the local reality. They may coincide or be interconnected, indicating priority actions to be implemented in the territories and supporting the operationalization of cooperative intelligence through training managers and local communities to use strategic information about conditions to reduce food insecurity [2,12,14]. This is particularly relevant considering the application of these principles within the scope of the Brazilian National School Feeding Program (Programa Nacional de Alimentação Escolar—PNAE) and the need for knowledge of the territory to implement Food and Nutritional Security (FNS).

Studies on food deserts and swamps around schools have been developing in Brazil. A survey of the food supply in public schools (Santos, São Paulo) classified the foods offered in food sales establishments, in terms of processing, into ultra-processed and minimally processed. The results indicated that, in the 500 m buffer of the school territory, the sale of ultra-processed foods predominated around the schools, which could lead to greater exposure of students to the acquisition and consumption of these foods of lower nutritional quality [18]. A study in Juiz de Fora, Minas Gerais evaluated the food environment in the school territory and showed agglomerations of all categories of food sales establishments around the schools. The authors found greater agglomeration and diversity of food establishments in the central region of the municipality, which was considered healthier than the peripheral region [11]. Another study evaluated the community food environment and the existence of food swamps around schools in Rio de Janeiro. It showed a predominance of food establishments selling ultra-processed foods, such as bars and cafeterias around schools, which exposes children and adolescents to an unhealthy food environment [16].

In Brazil, since 2010, when a legal framework defined FNS as a right, ways of identifying and measuring FNS have been discussed due to its multiple dimensions, which reflect different perspectives and purposes of use [19]. Kepple and Segall-Corrêa (2011) state that food security encompasses four essential dimensions. The first involves food availability for the population, depending on production, import, and distribution systems. The second concerns physical and economic access to food in sufficient quantity and with adequate nutritional quality, influenced by price policies and family income. The third dimension is related to the biological use of food, impacted by sanitary conditions and social habits. The fourth dimension is stability, which requires public and family actions to deal with chronic, seasonal, or temporary access problems, aiming for social, economic, and environmental sustainability [2].

In the context of the PNAE, the four dimensions discussed above can be applied to ensure students’ FNS at school and comprehend the dynamics of local food environments, including the availability of healthy and unhealthy foods in school canteens and around schools. It is essential to guarantee nutritious meals and promote healthy eating among students [2,20,21] since, based on identifying these characteristics, it is possible to think of specific assistance strategies to minimize food insecurity. By building networks of collaboration and information sharing, stakeholders can identify gaps in the supply of healthy foods, create intervention strategies, and improve the nutritional profile of school meals, thus contributing to the health and well-being of the student population, especially the most vulnerable [6].

In this sense, this study aims to map deserts, swamps and food oases around schools in Bahia, emphasizing the importance of implementing the PNAE in these areas, to guarantee food and nutritional security.

## 2. Materials and Methods

### 2.1. Study Design and Sample Characterization

This ecological study was performed in the census sectors comprising all the 35 public school units, composed of the IFBA and IFBAIANO campuses, considered important federal institutes (a kind of public school) in the State of Bahia. The buffer was the neighborhood unit used to evaluate the food environment in the territory. Taking each school as a central point, 800 m circular buffers were built since this distance is recurrent for this type of analysis [1].

All food sales establishments, formal and informal, existing in this perimeter were mapped to verify the food environment in general. Analyzing deserts, swamps, and food oases, the types of establishments were restricted according to the methodology adopted. School canteens were not considered since the school’s internal environment acts as an exclusive environment for consumers in that location but does not characterize the surrounding area.

### 2.2. Variables Related to Food and Nutritional Security and Vulnerability

Data on nutritional vulnerability were obtained through the technical document containing the update of territories with the highest levels of food and nutrition insecurity (FNI) in Brazil and the Survey on Food Insecurity and COVID-19 in Brazil [5,7]. The project called IICA/BRA created the social vulnerability index of the Brazilian population, in which microdata from the Brazilian Scale of Food and Nutritional Insecurity (EBIA) and the Personal Food Consumption of the Brazilian Population were combined to classify the population according to levels of food security and food insecurity (mild, moderate, severe, and very severe), based on the Food Insecurity Experience Scale (FIES) developed by FAO [5].

The Gini Index is an instrument to measure the degree of income concentration in a given group. It points out the difference between the poorest and the wealthiest incomes, varying from zero to one, where zero represents income equality and one is at the opposite extreme, meaning that one person holds all the wealth. In this study, the Gini Index is related to the population’s purchasing power and vulnerability depending on the concentration of income in the region analyzed [6].

Food consumption data were obtained through the Family Budget Survey (*Pesquisa de Orçamento familiar*—POF) 2017–2018 [22]. Information on student enrollment in schools was obtained through data from the 2022 School Census [5].

### 2.3. Data Geocoding

The geographic coordinates (latitude and longitude) of s and food establishments were initially obtained using the online search service Google Maps (https://www.google.com.br/maps?hl=pt-BR, accessed on 20 March 2022) and subsequently by active search around the s, with an actual marking of the location obtained. The data were finally collected in the WGS 84 Geographic Coordinate System configuration and subsequently transformed into the Projected Coordinate System, Universal Transverse Mercator System (UTM), 23S time zone, SIRGAS 2000 datum, through the use of the free software QGIS, version 3.30.2 (Brazil, 2021).

### 2.4. Characterization of Food Deserts and Swamps

Taking each public school as a central point, 800 m circular buffers were built. The buffer analysis corresponds to the methodological needs for identifying deserts, swamps, and food oases, as described in the following items.

The methodology proposed by the CDC was used to evaluate food deserts and swamps. According to this institution, food deserts refer to urban areas lacking physical access to essential foods, such as fruits, vegetables, whole grains, low-fat dairy products, and other items characteristic of a healthy diet. In contrast, swamp environments are defined as neighborhoods with a predominance of establishments that sell ultra-processed foods [23].

The Modified Retail Food Environment Index/mRFEI for the Brazilian reality was calculated following the proposal by Honório et al. (2021), in which all cafeterias were considered fast food restaurants and fresh produce markets were replaced by fruit and vegetable markets [13]. The formula described below was used.

$$\mathrm{mRFEI}=\frac{\mathrm{supermarkets}+\mathrm{hypermarkets}+\mathrm{fruit}\;\mathrm{and}\;\mathrm{vegetable}\;\mathrm{stores}}{\mathrm{cafeterias}+\mathrm{grocery}\;\mathrm{stores}}\times 100$$

The number of food establishments was counted within the 800 m buffer, considering the public school as centroid. The surrounding area was considered a food desert when the result was zero. When there was no establishment selling food within 800 m of the surrounding area, we adopted the name acute desert; when there was some establishment, but the mRFEI calculation was equal to zero, we adopted the term moderate desert for a more in-depth discussion on food and nutritional security.

When values between 0.01 and 20 were obtained for the mRFEI, the locations were classified as food swamps. Since the CDC does not present a cutoff point for food swamps, we adopted this cutoff following an analysis by Honório et al. (2021) [13]. For values above 20, the neighborhood was classified as mixed, indicating many establishments selling healthy and unhealthy foods.

### 2.5. Characterization of Food Oases

To characterize food oases, we used the methodology proposed by Walker et al. (2010), who introduced the concept of food oases (neighborhoods with a supermarket within 800 m of the zip code centroid) [12]. For this study, we adopted the same suggested distance from public school (centroid).

It is important to note that this classification can be combined with deserts, swamps, or mixed locations, indicating broad possibilities for the consumer, even within an environment with more prevalent characteristics of the previous groups.

### 2.6. Statistical Analysis

The data were inserted in a single spreadsheet and then grouped according to the analysis required for each proposed methodology. Descriptive analyses were carried out with frequency measurements, measures of central tendency (mean and median) and dispersion (standard deviation). The food environment of public schools was compared considering the number of students impacted, school location (urban or rural area) and the size of the municipalities. For this purpose, the statistical software SPSS 28.0.1 was used.

### 2.7. Ethical Aspects

The research project was approved by the Research Ethics Committee of the Federal University of Bahia, Brazil under registration CAAE: 57747322.5.0000.5023

## 3. Results and Discussion

### 3.1. Food Environment around Schools

The study was carried out considering the surroundings of the 35 schools in the state of Bahia. The analysis of the food environment around schools becomes relevant when considering that this environment impacts 49,494 enrolled students in these public schools, according to the 2022 School Census.

**Figure 1:**
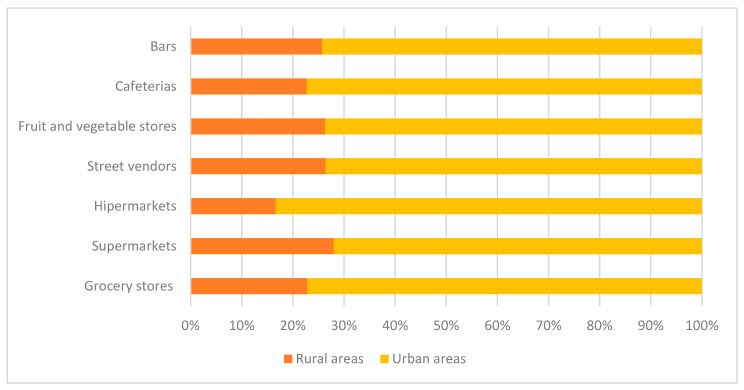
Percentage of establishments according to the location of public schools in Bahia (urban and rural areas), Brazil.

Concerning the food environment, 403 establishments that sell food or meals for immediate consumption were identified, of which 69.2% (*n* = 279) were formal food establishments and 30.8% (*n* = 124) were informal (not registered). The most common establishments around schools were cafeterias (32.7%, *n* = 132) and grocery stores (26%, *n* = 105). Figure 1 shows the percentages of all food establishments mapped around schools, comparing those in urban areas with those in rural areas.

The assessment of the community food environment around schools has gained emphasis, mainly because studies indicate that they influence the children’s and adolescents’ food choices [3,16,18] and can affect students’ health and quality of life [1,9,11,24].

Considering the buffer of 800 m, the average number of establishments that sell food or meals for immediate consumption was 22.39 (±13.03). By stratifying food establishments according to a category, cafeterias (7.33 ± 4.43), grocery stores (5.83 ± 4.09), and restaurants (2.94 ± 2.19) were more frequent. Furthermore, 80.0% (*n* = 28) of the s had at least one immediate consumption establishment within the 800 m buffer, with 71.4% of the buffers having at least one cafeteria, 77.1% having at least one grocery store, and 51.4% at least one restaurant.

A study analyzed all public and private schools (*n* = 1436) in Belo Horizonte, Brazil and observed that cafeterias (4.10 ± 7.50), restaurants (3.60 ± 5.70), and bars (2.80 ± 2.80) were the categories that had the highest averages around schools [17]. Another study carried out in Rio de Janeiro, Brazil analyzed the food environment around 3159 public and private schools and identified that coffeeshops, cafeterias, bars, and minimarkets were the most present food establishments around schools, with at least one establishment found nearby in more than 90% of schools analyzed [16]. These two studies present similarities with the present work concerning the type of food trade, emphasizing places selling industrialized products.

Bars represented the third most present category in the study by Peres et al. (2021) and were in fourth place in the study by Andretti et al. (2023) as well as in this study, in which bars also occupied fourth place in schools’ food environment, making up a percentage of 8.7% (*n* = 35). Although these three studies were carried out in different locations in Brazil, with different consumption and income data, the presence of bars occupying a prominent position in all of them is a point of attention, as, in addition to being known for selling ready-to-eat foods with low nutritional value, according to the Family Budget Survey (POF) 2017–2018 [22], beer (65.4%) and wine (49.9%) are the items with the highest percentage of consumption outside the home among adolescents, often being related to the phase of rebellion, self-affirmation and contestation of imposed rules [16,17,25]. 

In Brazil, according to data from the Interministerial Chamber of Food and Nutritional Security (CAISAN), which carried out a mapping study of food deserts in Brazil, cafeteria are the main places for selling ultra-processed foods [14] and data from POF 2017–2018 on food consumption by teenagers corroborates this information: sandwich cookies, soft drinks, snacks, sandwiches and pizzas make up around 65% of food purchases outside the home by teenagers [22]. Furthermore, research that evaluated the food environment close to schools identified most foods of low nutritional quality in cafeterias, thus exposing children and adolescents to an obesogenic food environment with a high health risk, such as food swamps and mixed environments [1,9,16,17,18], which were also identified in the present study.

Of the 35 public schools evaluated, 19 (54.3%) were located in the urban area of the municipalities, while 16 (45.7%) were located in the rural area. Furthermore, considering the distance between the school and the city center, an average of 4.7 km and a median of 3.9 km were found, varying from 0.5 km to 17 km, which could represent a significant difference in the distribution space of commercial establishments, especially in the food sector.

The predominant presence of food establishments in the central areas of cities can occur due to the high number of people in these urban regions but not just due to the presence of schools in these locations. A study showed a concentration of establishments that sell food close to schools, and, in turn, these schools tend to be located in areas with greater movement of people [18]. Additionally, the authors observed that the density of food stores around schools decreased as the socioeconomic vulnerability of the neighborhood increased. A study corroborates these data, providing information that, in Rio de Janeiro, Brazil, the concentration of food establishments, in general, occurred closer to areas with high circulation and greater purchasing power of the local population, with a significant reduction in the density of establishments in regions with the lowest tercile of income [16]. This suggests a connection between the location of schools, the availability of food and the financial situation of residents, possibly due to the lower attractiveness of the most vulnerable districts for the installation of food establishments due to precarious infrastructure, high crime rates and lower purchasing power of residents [26].

Regarding the degrees of vulnerability, according to data on the levels of Food and Nutritional Insecurity released by IICA in 2023 [7], 68.6% (*n* = 24) of the schools analyzed are located in regions of medium vulnerability, 28.6% (*n* = 10) in areas of high vulnerability and only one school (2.8%) in a region of very high vulnerability. Associated with these data, we have the GINI coefficient with an average of 0.566 (±0.035) in the municipalities where the s are located, indicating a high degree of inequality in the income distribution in these locations.

Consequently, students can develop different interactions with the food environment close to their schools, depending on the level of vulnerability of the neighborhood in which they are located. While those who attend schools in vulnerable areas may have limited access to places to purchase ready-to-eat foods, those who study in more developed regions, especially in the city center, are exposed to various food establishments, but these places do not always offer many options for healthy food. Research suggests that a lack of choice when purchasing and consuming healthy foods may be associated with harmful health effects [9,25].

### 3.2. Deserts, Swamps and Food Oases around Schools

In the context of the schools presented in this work, 7 are in regions of food swamps, 14 in food deserts and 23 in food oases, and 14 schools have food establishments in their surroundings that provide both healthy and processed/ultra-processed foods (mixed neighborhoods) (Table 1). Studies establishing methodologies for identifying food deserts and swamps around schools are still consolidating in Brazil, but it is already possible to see an awakening to the importance of this topic [16,17,26].

The two extremes represented by deserts and food swamps proved to be a warning sign, as they impact 64.2% of all students studying at public schools in Bahia (*n* = 31,773). Regarding the location of these neighborhoods, it is possible to observe a clear distinction between urban and rural areas, with a greater concentration of deserts (especially acute ones) in rural areas, while swamps are concentrated in urban areas. Among the 16 schools located in rural areas, 3 (19%) are greater than 10 km from the urban center and this can represent a significant impact on the installation of food retail establishments, as they depend on the flow of people, raw materials and services to make a profit. A Brazilian study carried out in 2023 reflects on the worrying presence of food deserts in the countryside, related to increased unemployment, low income, low education, gender, race/color and difficulty in accessing public transport, among others [27].

A high prevalence of food deserts was identified, with acute deserts found in most buffers. This configuration mainly occurred in schools located in rural areas and medium-sized cities. Andretti et al. (2023) reported that 15% of the schools analyzed were located in neighborhoods categorized as food deserts, with the highest prevalence around public schools, low-income populations and high food insecurity conditions [16]. Peres et al. (2021), despite not having a specific methodology for identifying food deserts, found that 37 schools (2.58%) did not have any immediate consumption establishments in their surroundings [17]. Our study showed a higher percentage of food deserts than the previously mentioned studies performed in Brazil. This is probably due to the analysis being carried out in large urban centers, unlike the locations that comprise the sample of the present study, the majority of which are concentrated in the interior of Bahia, with only three schools located in the capital and metropolitan region of the state.

Considering the availability of unhealthy food establishments in the evaluated buffers, 20% of schools were located in a buffer classified as a food swamp, mainly in the urban zone of medium and large cities and impacting 30% of the total number of students enrolled. Peres et al. (2021) found that 54.6% of schools were located in food swamps, with the highest density around private high schools and higher-income census tracts [17]. Andretti et al. (2023) identified the presence of food swamps in 97% of the neighborhoods analyzed, and they are also more prevalent around private high schools and in the highest-income tertiles [16]. Also, regarding food swamps, one can notice the disparity between the location of the school in the urban area and the population’s income concentration.

Despite representing the lowest percentage among the groups analyzed, the influence of food swamps on the population’s eating habits cannot be ignored, as they strongly correlate with negative health outcomes. In Brazil, the sale of ultra-processed foods in cafeterias and bars is predominant [14] and studies carried out with Brazilian children and adolescents have shown that the purchase of food in these establishments had a positive correlation with an increase in the intake of ultra-processed foods [10,11,25,28,29]. Furthermore, studies that analyzed the food environment around schools in several countries found that the foods sold had, in general, low nutritional quality, thus exposing children and adolescents to an obesogenic food environment [6,9,24,28].

Table 1 shows a significant percentage (40%) of schools located in an environment considered mixed, with a large concentration of healthy and unhealthy establishments. In Rio de Janeiro, Brazil, 12% of neighborhoods were characterized as either food deserts or food swamps, correlating with low income, high deprivation, and high economic segregation [16]. These data reinforce the idea that socioeconomic distribution influences food environments around schools, despite no consensus in the literature [1,3,17]. Mixed neighborhoods can also be characterized as obesogenic environments, as they are marked by the presence of unhealthy foods for immediate consumption, and are very important when discussing food and nutritional security, especially for the adolescent population, a focus taken in this work [18].

Filgueiras et al. (2023) conducted research in the state of Minas Gerais, Brazil to identify elements of the environment close to schools that could have an impact on obesity and the inflammation associated with it. Aspects such as the food environment, environment for practicing physical activities and factors related to safety and crime in the city were included in the analysis. The authors reported that the density of establishments that sell ultra-processed foods demonstrated a positive association with total body fat and android body fat of children, in addition to the interference of factors such as spaces for physical activity, security and crime in concentrations of pro-inflammatory adipokines, such as leptin, adiponectin and RBP4 [11]. 

A systematic review study with meta-analysis highlighted that the sale of food at or near schools significantly increases the risk of obesity, while the availability of healthy food provided by the school significantly reduces this risk [30]. Other studies have also reported that the availability of unhealthy foods and drinks at school is linked to inadequate eating patterns [3,8,16,18,31] and that the consumption of ultra-processed foods, such as sweets, soft drinks and snacks, negatively impacts students’ weight [3,8,30], confirming that the food environment has a strong influence on the eating behavior and overweight/obesity of children and adolescents who are in this environment [3,9,28,30].

The increase in the consumption of unhealthy foods, with diets rich in fats and sugars indicates significant health concerns regarding food and nutritional security. This concern, which was already part of the Brazilian context, was worsened after the COVID-19 pandemic, with worse health indicators, food inequality reduced access to healthy foods and low-quality diets, culminating in Brazil’s return to the world hunger map [5,7,19]. To solve these problems, it is imperative to discuss the current regulations for food sovereignty in Brazil, as well as the implementation of public policies to meet the needs of the vulnerable population, which includes students in Brazilian public schools.

Although food oases are still considered a new classification and without consolidated methodological parameters for their identification, they represent the highest percentage index among the four categories, presenting us with an interesting perspective regarding possibilities. Supermarkets and hypermarkets offer a range of products for consumers, who also need to have gone through a food and nutritional education process, but who can find excellent food options, with different degrees of processing and thus compose a healthy diet. A study conducted in Texas identified a greater number of healthy foods, composed of low fat and calorie content and high composition of whole grains in food oasis regions compared to the others [15]. Concerning foods for immediate consumption, supermarkets represent a good alternative, as it is possible to purchase fresh fruits, both fresh and minimally processed, dairy products and oilseeds, which can make up a nutritionally balanced snack. However, these places are also marked by the sale of ready-to-eat industrialized and ultra-processed products, such as cookies, snacks, fried foods, sweets and treats, as well as a huge variety of sugary drinks, generally preferred by adolescents [8,32].

### 3.3. Impacts Related to Food and Nutritional Security and Importance of the National School Feeding Program (Programa Nacional de Alimentação Escolar—PNAE)

The II National Survey on Food Insecurity in Brazil, carried out in 2022, provided worrying data about the situation in the country, in which 125.2 million people are in food insecurity and more than 33 million are in a situation of hunger (severe food insecurity) [5]. In the state of Bahia, where the schools analyzed are located, 62.6% of the population is in food insecurity, with 11.4% suffering from severe food insecurity [5,7]. These data reveal that food insecurity affects the rural population more significantly than the urban population and that this situation was profoundly worsened after the COVID-19 pandemic. This is due to many factors, including challenges in transporting agricultural products, high prices, significant production losses and decreased supportive public policies [7,19].

Furthermore, studies demonstrated that food insecurity was around 25% higher in households where three or more people aged up to 18 lived, revealing that the presence of children and adolescents in families is related to the greater severity of food insecurity in the country [5]. Food insecurity and hunger among children and adolescents have been the subject of recent studies, and data from the United Nations International Children’s Emergency Fund (UNICEF) reveal the negative and immediate effects on their health and well-being conditions and warn of future impacts that compromise these young people’s physical and social potential [6].

The convergence of these studies allows us to bring to light the reflection on the lack of food choices for these individuals, widening the abyss concerning nutrition and health conditions and indirectly impacting the school’s interference on this nutritional issue, as it can be configured as an environment in which students can find adequate nutritional support, at least during the period in which they are studying, through existing nutrition programs within the school. The denial of rights goes against the concept of food and nutritional security, resulting in a lack of constant access to healthy and sustainable food, disrespecting environmental, economic, cultural and social sustainability and harming food sovereignty in the territory [7,19].

As the object of our study is the public basic education in schools, one of the main resources for guaranteeing FNS for students is the implementation of the Brazilian school meal program (PNAE) in these institutions to ensure the right to adequate and healthy food for students and to meet their daily nutritional needs, following the assumptions of current legislation [33].

The PNAE is a public policy implemented in 1955 to contribute to students’ growth, development, learning, academic performance and healthy eating habits through providing school meals and interventions related to nutritional education. According to current PNAE legislation, priority is given to fresh and minimally processed foods. Thirty percent of all resources allocated to the PNAE must be family farming products, and the menu must offer three portions of fruit and vegetables per week (200 g/student/week). On the other hand, ultra-processed products such as canned goods, sausages, drinks with low nutritional value and sweets are prohibited or restricted on the school menu legislation [33]. Thus, providing school meals in public schools, combined with lower sales and advertising of processed foods within schools, can generate a healthier food environment and lead to better food consumption among students, reducing the need to purchase food in the school environment and, consequently, the harmful effects on students’ health [20,21].

Recent studies showed that, in general, there is less consumption of sugar, sugary drinks and processed and ultra-processed foods among students in public schools compared to private schools [16,17,30]. Some Brazilian studies have highlighted the benefits of school meals offered in Brazilian public schools for healthy eating practices, highlighting the importance of the PNAE in Brazil [8,18,34]. A study performed in Belo Horizonte, Brazil in 2018 with 1357 students from public schools revealed that students who consumed two or three school meals a day had a 7.3% higher consumption of fresh foods and 10.5% higher consumption of minimally processed foods. Furthermore, these students consumed 18% and 26% less ultra-processed foods when compared to those who did not consume school meals [34].

According to PNAE, part of the dietitians’ duties is to carry out food and nutrition education (FNE) activities, which can offer students technical knowledge about the composition of foods and nutritional labeling, as well as tools to make daily choices more balanced [20,21,33]. Furthermore, Brazilian legislation includes the topic of FNE as a transversal topic in the school curriculum [35] to minimize the effects of the lack of knowledge on students’ health. In adolescents, there is a frequent discrepancy between their knowledge about healthy eating and their eating reality. This reality is often marked by a sequence of nutritionally deficient choices, influenced by the environment in which they live, the media, family eating habits or the search for autonomy concerning previously imposed standards [30]. Carrying out FNE at school can promote responsibility in adolescents regarding food choices suited to their needs, empower them about the integrity of the information conveyed in the various media to which they are subject daily, as well as train them to carry out better choices given the food environment they are exposed to daily.

Methodological limitations were identified, as the studies are still heterogeneous and there is no established standard for analyzing deserts, swamps and food oases in Brazilian territory, nor around schools. Furthermore, few studies have been carried out on this topic in Brazil, limiting the comparison with data from other authors and highlighting the need for more research to expand knowledge about deserts, swamps and food oases, their influence on students’ choices, as well as the consequences of these environments on their health and quality of life. Another limitation may refer to the possibility of purchasing food in surrounding areas and on the Bahia’s borders, as is the case of Juazeiro. Furthermore, there is difficulty in demarcating buffer locations, as some geographic regions are often shared.

## 4. Conclusions

It was possible to highlight through this work the marked presence of deserts, swamps and food oases around schools in Bahia, Brazil and their relationship with the size of the municipalities, the location of the school in an urban or rural area and the distance from the school to the center city. This context was connected to food and nutritional insecurity, which has taken on greater proportions in Brazil and directly affects the study population.

Georeferencing studies have represented important mechanisms for understanding the organization of spaces and obtaining data related to eating behavior in different contexts, with public schools being the focus of this work. Although it was not the object of this study, knowing the maps and geographic data of the space can provide important data that strengthens the discussion about the food environment. This work highlighted the importance of implementing PNAE in schools, by providing adequate and balanced food that meets students’ nutritional needs, guaranteeing food security and promoting health through food and nutritional education, which improves students’ eating habits and nutritional status. Finally, promoting an environment that favors the supply and choice of nutritious foods and strengthening the PNAE should be management priorities to reduce the current high levels of food insecurity among the population and contribute to the promotion of healthy eating for students. Further studies are needed to demonstrate how spatial organization influences food choices and the findings of this study can serve as a basis for further research to highlight the food environment at a national level.

## Figures and Tables

**Table 1:** Classification of food environment considering schools location, size of municipalities and students impacted.

	Acute Desert	Moderate Desert	Swamp	Mixed	Oases	Total
**Schools** **% (*n*)**	28.6% (*n* = 10)	11.4% (*n* = 4)	20.0% (*n* = 7)	40.0% (*n* = 14)	65.7% (*n* = 23)	100% (*n* = 35)
**Impacted students** **% (*n*)**	22.6% (*n* = 11.184)	11.6% (*n* = 5.755)	30% (*n* = 14.834)	35.8% (*n* = 17.721)	70.5% (*n* = 34.902)	100% (*n* = 49.494)
**School’s location**						
**Urban area** **% (*n*)**	20% (*n* = 2)	50% (*n* = 2)	71.4% (*n* = 5)	28.6% (*n* = 4)	65.2% (*n* = 15)	54.3% (*n* = 19)
**Rural area** **% (*n*)**	80% (*n* = 8)	50% (*n* = 2)	28.6% (*n* = 2)	71.4% (*n* = 10)	34.8% (*n* = 8)	45.7% (*n* = 16)
**Size of municipalities**						
**Small cities ¹**	20% (*n* = 2)	0	0	7.1% (*n* = 14)	4.3% (*n* = 1)	8.6% (*n* = 3)
**Medium-sized cities ^2^**	60% (*n* = 6)	75% (*n* = 3)	28.6% (*n* = 2)	28.6% (*n* = 14)	30.5% (*n* = 7)	42.8% (*n* = 15)
**Large cities ^3^**	20% (*n* = 2)	25% (*n* = 1)	71.4% (*n* = 5)	64.3% (*n* = 9)	65.2% (*n* = 15)	48.6% (*n* = 17)

^1^ Municipalities with up to 50,000 inhabitants; ^2^ municipalities with up to 100,000 inhabitants; ^3^ municipalities with up to 900,000 inhabitants.

## Data Availability

Data are contained within the article.

## References

[1] França F.C.O.d., Andrade I.d.S., Zandonadi R.P., Sávio K.E., Akutsu R.d.C.C.d.A. (2022). Food Environment around Schools: A Systematic Scope Review. Nutrients.

[2] Kepple A.W., Segall-Corrêa A.M. (2011). Conceptualizing and Measuring Food and Nutrition Security. Cienc. Saude Coletiva.

[3] Matsuzaki M., Sanchez B., Acosta M., Botkin J., Sanchez-Vaznaugh E. (2020). Food Environment near Schools and Body Weight-A Systematic Review of Associations by Race/Ethnicity, Gender, Grade, and Socio-Economic Factors. Obes. Rev..

[4] Marien Da Costa Peres C., Soares Gardone D., Vieira De Lima Costa B., Kü Mmel Duarte C., Pessoa C., Mendes L.L. (2020). Retail Food Environment around Schools and Overweight: A Systematic Review. Nutr. Rev..

[5] Rede PENSSAN (2022). II VIGISAN: Inquérito Nacional Sobre Insegurança Alimentar No Contexto da Pandemia da COVID-19 No Brasil.

[6] UNICEF (2021). Impactos Primários e Secundários da COVID-19 em Crianças e Adolescentes Relatório de Análise 2a Rodada.

[7] Instituto Interamericano de Cooperação para a Agricultura (2023). Projeto bra/iica/17/001 Segurança Alimentar e Nutricional: A Disponibilidade e o Acesso à Alimentos Saudáveis e o Combate à Pobreza Rural.

[8] Azeredo C.M., de Rezende L.F.M., Canella D.S., Claro R.M., Peres M.F.T., Luiz O.d.C., França-Junior I., Kinra S., Hawkesworth S., Levy R.B. (2016). Food Environments in Schools and in the Immediate Vicinity Are Associated with Unhealthy Food Consumption among Brazilian Adolescents. Prev. Med..

[9] Day P.L., Pearce J. (2011). Obesity-Promoting Food Environments and the Spatial Clustering of Food Outlets Around Schools. Am. J. Prev. Med..

[10] Correa E., Rossi C., das Neves J., Silva D., de Vasconcelos F. (2018). Utilization and Environmental Availability of Food Outlets and Overweight/Obesity among Schoolchildren in a City in the South of Brazil. J. Public Health.

[11] Filgueiras M.D.S., Pessoa M.C., Bressan J., Fogal Vegi A.S., do Carmo A.S., de Albuquerque F.M., Gardone D.S., de Novaes J.F. (2023). Characteristics of the Obesogenic Environment around Schools Are Associated with Body Fat and Low-Grade Inflammation in Brazilian Children. Public Health Nutr..

[12] Walker R.E., Butler J., Kriska A., Keane C., Fryer C.S., Burke J.G. (2010). How Does Food Security Impact Residents of a Food Desert and a Food Oasis?. J. Hunger Environ. Nutr..

[13] Honório O.S., Horta P.M., Pessoa M.C., Jardim M.Z., do Carmo A.S., Mendes L.L. (2021). Food Deserts and Food Swamps in a Brazilian Metropolis: Comparison of Methods to Evaluate the Community Food Environment in Belo Horizonte. Food Secur..

[14] Câmara Interministerial de Segurança Alimentar e Nutricional (CAISAN) (2018). Mapeamento dos Desertos Alimentares No Brasil.

[15] Jin H., Lu Y. (2021). Evaluating Consumer Nutrition Environment in Food Deserts and Food Swamps. J. Environ. Res. Public Health.

[16] Andretti B., Cardoso L.O., Honório O.S., de Castro Junior P.C.P., Tavares L.F., da Costa Gaspar da Silva I., Mendes L.L. (2023). Ecological Study of the Association between Socioeconomic Inequality and Food Deserts and Swamps around Schools in Rio de Janeiro, Brazil. BMC Public Health.

[17] da Costa Peres C.M., de Lima Costa B.V., Pessoa M.C., Honório O.S., do Carmo A.S., da Silva T.P.R., Gardone D.S., Meireles A.L., Mendes L.L. (2021). Community Food Environment and Presence of Food Swamps around Schools in a Brazilian Metropolis. Cad. Saude Publica.

[18] Leite M.A., De Assis M.M., Do Carmo A.S., Da Silva T.P.R., Nogueira M.C., Netto M.P., Levy R.B., Mendes L.L. (2021). Disparities in Food Availability around Schools in a Large Brazilian City. Child. Youth Environ..

[19] Instituto Brasileiro de Geografia e Estatística (2020). Pesquisa de Orçamentos Familiares 2017–2018: Análise da Segurança Alimentar No Brasil.

[20] Lima D.R.d.S., Diogo S.S., Peixinho A.M.L., Cabrini D. (2023). Programa Nacional de Alimentação Escolar (PNAE): Marcos Históricos, Políticos e Institucionais Que Influenciaram a Política Nos Seus Quase 70 Anos de Existência. Rev. Alim. Cult. Am..

[21] De Rezende L.T., Gottschall L.M., da Silva Sampaio K.P., de Freitas Castro S.F. (2022). Avanços Da Legislação Do Programa Nacional Da Alimentação Escolar. Cad. FNDE.

[22] IBGE—Instituto Brasileiro de Geografia e Estatística (2020). Pesquisa de Orçamentos Familiares 2017–2018: Análise do Consumo Alimentar Pessoal No Brasil.

[23] CDC (2011). Census Tract Level State Maps of the Modied Retail Food Environment Index (MRFEI).

[24] Do Carmo A.S., de Assis M.M., Cunha C.d.F., de Oliveira T.R.P.R., Mendes L.L. (2018). The Food Environment of Brazilian Public and Private Schools. Cad. Saude Publica.

[25] Gubbels J.S. (2020). Environmental Influences on Dietary Intake of Children and Adolescents. Nutrients.

[26] Duran A.C., De Almeida S.L., Latorre M.D.R.D., Jaime P.C. (2016). The Role of the Local Retail Food Environment in Fruit, Vegetable and Sugar-Sweetened Beverage Consumption in Brazil. Public Health Nutr..

[27] Guimarães N.A., Matielo E. (2023). Reflexões sobre a Instalação de Desertos Alimentares no Campo Brasileiro. Cardernos OBHA.

[28] Rocha L.L., Gratão L.H.A., do Carmo A.S., Costa A.B.P., de Freitas Cunha C., de Oliveira T.R.P.R., Mendes L.L. (2021). School Type, Eating Habits, and Screen Time Are Associated with Ultra-Processed Food Consumption Among Brazilian Adolescents. J. Acad. Nutr. Diet.

[29] Bezerra I.N., Moreira T.M.V., Cavalcante J.B., de Moura Souza A., Sichieri R. (2017). Food Consumed Outside the Home in Brazil According to Places of Purchase. Rev. Saude Publica.

[30] Gonçalves V.S.S., Figueiredo A.C.M.G., Silva S.A., Silva S.U., Ronca D.B., Dutra E.S., Carvalho K.M.B. (2021). The Food Environment in Schools and Their Immediate Vicinities Associated with Excess Weight in Adolescence: A Systematic Review and Meta-Analysis. Health Place.

[31] Barata M., Leite M., Levy R. (2020). The Food Environment in School’s Vicinities of Sao Paulo, Brazil. Eur. J. Public Health.

[32] Instituto Brasileiro de Geografia e Estatística (2020). Pesquisa de Orçamentos Familiares 2017–2018: Avaliação Nutricional da Disponibilidade Domiciliar de Alimentos No Brasil.

[33] Brasil. Fundo Nacional de Desenvolvimento da Educação. FNDE. Lei No 11.947, de 16 de Junho de 2009. http://www.planalto.gov.br/ccivil_03/_ato2007-2010/2009/lei/l11947.htm.

[34] Bento B.M.A., Moreira A.d.C., do Carmo A.S., dos Santos L.C., Horta P.M. (2018). A Higher Number of School Meals Is Associated with a Less-Processed Diet. J. Pediatr..

[35] (2018). Brasil. Presidência da República. Lei No 13.666, de 16 de Maio de 2018. Altera a Lei No 9.394, de 20 de de- Zembro de 1996 (Lei de Diretrizes e Bases Da Educação Nacional), Para Incluir o Tema Transversal Da Educação Alimentar e Nutricional No Currículo Escolar. *Diário of União*. https://legislacao.presidencia.gov.br/atos/?tipo=LEI&numero=13666&ano=2018&ato=495ETU61UeZpWTe25.

